# The NOTCH3 score: a pre-clinical CADASIL biomarker in a novel human genomic *NOTCH3* transgenic mouse model with early progressive vascular NOTCH3 accumulation

**DOI:** 10.1186/s40478-015-0268-1

**Published:** 2015-12-29

**Authors:** Julie W. Rutten, Roselin R. Klever, Ingrid M. Hegeman, Dana S. Poole, Hans G. Dauwerse, Ludo A. M. Broos, Cor Breukel, Annemieke M. Aartsma-Rus, J. Sjef Verbeek, Louise van der Weerd, Sjoerd G. van Duinen, Arn M. J. M. van den Maagdenberg, Saskia A. J. Lesnik Oberstein

**Affiliations:** Department of Human Genetics, Leiden University Medical Center, Leiden, The Netherlands; Department of Pathology, Leiden University Medical Center, Leiden, The Netherlands; Department of Radiology, Leiden University Medical Center, Leiden, The Netherlands; Department of Clinical Genetics, K5-R, Leiden University Medical Center, PO Box 9600, 2300 RC Leiden, The Netherlands; Department of Neurology, Leiden University Medical Center, Leiden, The Netherlands

**Keywords:** CADASIL, NOTCH3, Transgenic mouse model, Biomarker

## Abstract

**Introduction:**

CADASIL (Cerebral Autosomal Dominant Arteriopathy with Subcortical Infarcts and Leukoencephalopathy) is a hereditary small vessel disease caused by mutations in the *NOTCH3* gene, leading to toxic NOTCH3 protein accumulation in the small- to medium sized arterioles. The accumulation is systemic but most pronounced in the brain vasculature where it leads to clinical symptoms of recurrent stroke and dementia. There is no therapy for CADASIL, and therapeutic development is hampered by a lack of feasible clinical outcome measures and biomarkers, both in mouse models and in CADASIL patients. To facilitate pre-clinical therapeutic interventions for CADASIL, we aimed to develop a novel, translational CADASIL mouse model.

**Results:**

We generated transgenic mice in which we overexpressed the full length human *NOTCH3* gene from a genomic construct with the archetypal c.544C > T, p.Arg182Cys mutation. The four mutant strains we generated have respective human *NOTCH3* RNA expression levels of 100, 150, 200 and 350 % relative to endogenous mouse *Notch3* RNA expression. Immunohistochemistry on brain sections shows characteristic vascular human NOTCH3 accumulation in all four mutant strains, with human *NOTCH3* RNA expression levels correlating with age at onset and progression of NOTCH3 accumulation. This finding was the basis for developing the ‘NOTCH3 score’, a quantitative measure for the NOTCH3 accumulation load. This score proved to be a robust and sensitive method to assess the progression of NOTCH3 accumulation, and a feasible biomarker for pre-clinical therapeutic testing.

**Conclusions:**

This novel, translational CADASIL mouse model is a suitable model for pre-clinical testing of therapeutic strategies aimed at delaying or reversing NOTCH3 accumulation, using the NOTCH3 score as a biomarker.

**Electronic supplementary material:**

The online version of this article (doi:10.1186/s40478-015-0268-1) contains supplementary material, which is available to authorized users.

## Introduction

Cerebral Autosomal Dominant Arteriopathy with Subcortical Infarcts and Leukoencephalopathy (CADASIL) is a hereditary small vessel disease caused by mutations in the *NOTCH3* gene, leading to mid-adult onset stroke and dementia [1]. CADASIL is characterized by accumulation of the extracellular domain of the NOTCH3 protein (NOTCH3^ECD^) in the media of small- to medium-sized arterioles [2]. In addition, electron dense deposits (granular osmiophilic material, GOM) are seen in close vicinity to the vascular smooth muscle cells (VSMCs) [3]. The arteriopathy is systemic but most pronounced in the brain where it leads to degeneration of VSMCs [3] and a disturbed cerebral blood flow regulation [4]. This causes recurrent ischemic strokes and cognitive decline, starting at a mean age of 45–50 years [5]. To date, there is no therapy to prevent or delay symptoms in CADASIL.

NOTCH3 targeting therapies are in the pre-clinical phase of development (Rutten et al., unpublished, patent no. WO 2010085151 A2). The hitherto available CADASIL mouse models have important limitations with respect to their feasibility for testing such therapeutic strategies. Available models include transgenic models overexpressing human *NOTCH3* from a cDNA construct [6–8] or rat *Notch3* from a genomic construct [9], and models in which a mutation was introduced into the endogenous *Notch3* gene [10, 11]. The first and often only sign of CADASIL in these models is the presence of NOTCH3 accumulation in the vasculature [12], and in all human *NOTCH3* transgenic models, the NOTCH3 accumulation only becomes apparent at a high age [6–8]. Only the mouse model that expresses mutant rat Notch3 protein from a genomic DNA construct shows early onset vascular Notch3 accumulation with subsequent development of brain parenchymal lesions [9]. However, this model is less suitable as a translational CADASIL model due to the species difference, which creates an additional hurdle in bringing therapeutic compounds to clinical trials. For example, this would be the case for antisense therapeutic strategies targeting mutated pre-mRNA, a therapeutic approach which is being developed for increasing numbers of CNS disorders [13].

For therapeutic development, feasible clinical outcome measures and biomarkers are imperative, both in mouse disease models and in patients. In CADASIL patients, the variability in age at onset and progression of clinical symptoms, including the major symptoms of stroke and cognitive decline, limits their use as an outcome measure in clinical trials, because of the large number of patients that would have to be included to detect a treatment effect within a typical trial-timeframe of 2 years [14]. White matter lesions, detected on T2 weighted brain MRI images, are present prior to the onset of clinical symptoms and correlate with disease severity [15], but are not a reliable predictor of disease progression [16]. Changes in magnetic resonance diffusion histograms are a better predictor of disease progression, but have only been studied in symptomatic patients [16, 17]. Ideally, CADASIL therapies would be initiated in the pre-symptomatic disease phase, i.e. in young adults with a proven familial *NOTCH3* mutation. Vascular NOTCH3 protein accumulation could be an interesting therapeutic biomarker for CADASIL, as increased vascular NOTCH3 staining and GOM are consistently found in skin arterioles of pre-symptomatic patients, decades before onset of stroke and cognitive decline [18, 19].

In this study, we set out to generate a novel, translational CADASIL mouse model and to develop a relevant biomarker in this model. We generated a series of human *NOTCH3* transgenic mouse strains, with various expression levels of mutant *NOTCH3*. These mice develop cerebrovascular NOTCH3 accumulation characteristic of CADASIL at an early age, and the *NOTCH3* expression level correlates with both the age at onset and progression of vascular NOTCH3 accumulation. We developed a quantitative measure for the vascular NOTCH3 accumulation load, which we show to be a sensitive and robust biomarker for CADASIL in these mice.

## Materials and methods

### Generation of *NOTCH3* transgenic mice

**Fig. 1:**
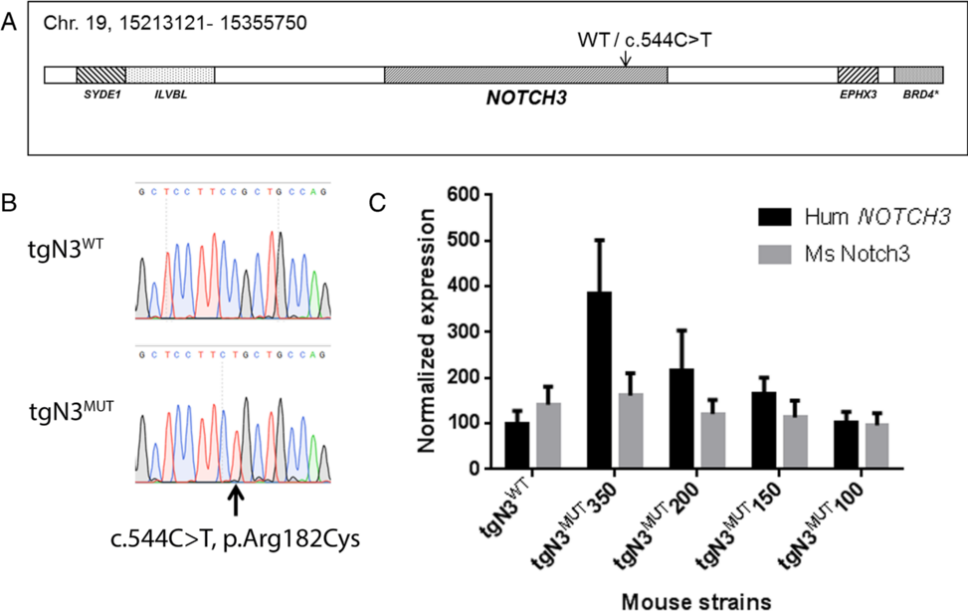
Generation of transgenic human *NOTCH3* mice. **a** Schematic representation of the BAC construct containing the human *NOTCH3* gene and flanking regions, used for generation of tgN3^WT^ and tgN3^MUT^ (c.544C > T, p.Arg182Cys) mice. **b** Sequencing analysis of PCR products of the human *NOTCH3* gene in transgenic mice confirmed the presence of the c.544C > T mutation in tgN3^MUT^ mice. **c** qPCR analysis of human and mouse *NOTCH3* expression in brain. In strain tgN3^WT^, human *NOTCH3* expression was comparable to endogenous mouse *Notch3* expression. The four mutant strains showed human *NOTCH3* expression levels of 350, 200, 150 and 100 %, as compared to endogenous mouse *Notch3* expression. Endogenous mouse *Notch3* expression was comparable between the transgenic mouse strains

For transgenesis, a 142,63 kb BAC clone was used (RP11-456 N16 BAC, Bacpac resources, Oakland, USA) (Ensemble release 59). The BAC contains the full-length human genomic *NOTCH3* gene and 44 kb of upstream and 67 kb of downstream sequence, including flanking genes *SYDE1*, *ILVBL*, *EPHX3* and a part of the *BRD4* gene (Fig. 1a). The c.544C > T (p.Arg182Cys) mutation was introduced using two-step Red-mediated recombination as previously described [20]. BAC constructs were injected into fertilized C57BL/6 J Ico oocytes. Positive transgenic founder mice were identified by PCR on DNA isolated from mouse ears using human specific primers (for primer sequences see Additional file 1: Table S1). The presence of the mutation was confirmed by direct Sanger sequencing analysis of PCR products (Fig. 1b). Five transgenic mouse strains were generated: one carrying the wild-type *NOTCH3* transgene (tgN3^WT^) and four carrying the mutant *NOTCH3* transgene (tgN3^MUT^). In each strain, integration of the BAC was confirmed by PCR analysis of *NOTCH3* and the flanking genes *SYDE1*, *ILVBL* and *EPHX3* (for primer sequences see Additional file 1: Table S1). All transgenic mouse strains bred normally. All experiments described in this study were approved by the local ethical committee for animal experimentation.

### *NOTCH3* expression analysis in *NOTCH3* transgenic mice

Total RNA was extracted from a brain hemisphere using RNA-Bee (Tel-test Inc., Friendswood, USA). For RT-PCR analysis, first-strand cDNA was synthesized using oligo (dT) primers. RT-PCR analysis was performed with primers across the human *NOTCH3* transcript (exons 2–4, exons 14–16, exons 30–32, exons 32–33, and the 3′UTR). For qPCR analysis, cDNA synthesis was performed with random hexamer primers, using the Revert Aid H Minus first strand kit (Thermo Scientific, Waltham, USA). Quantitative PCR was performed in four 10-week-old male and female transgenic mice and non-transgenic littermates, using both human- (exons 7–9, 216 bps) and mouse-specific (exons 6–8, 220 bps) primers. Mouse *Gapdh* was used as a reference gene, and human *NOTCH3* expression levels were calculated relative to endogenous mouse *Notch3* expression levels. A possible effect of differences in primer efficiencies of human- and mouse-specific *NOTCH3/Notch3* primersets was excluded by LinregPCR [21].

### NOTCH3 immunohistochemistry and quantification in *NOTCH3* transgenic mice

Vascular NOTCH3 accumulation in brain was analysed prospectively in groups of three mice, at age 4, 6, 12, 24, 52 and 82 weeks. In addition, vascular NOTCH3 accumulation was assessed in heart, aorta, liver, kidney, skin and tail at age 20 months. NOTCH3 immunohistochemistry was performed on cryosections that were fixated in acetone and incubated overnight with an antibody directed against the human NOTCH3^ECD^ (Novus Biologicals, Littleton, USA; dilution 1:2000). The following day, sections were incubated with peroxidase labelled polymer conjugated to anti-rabbit immunoglobulins (Envision kit, Dako, Glostrup, Denmark), and developed using 3,3′-diaminobenzidine. Quantification of the vascular NOTCH3 accumulation load was performed on brain cryosections. Per time point (6, 24, 52 and 82 weeks), three tgN3^MUT^350 mice and three tgN3^MUT^150 mice were analysed. For each mouse, four frontal lobe brain sections were NOTCH3 immunostained simultaneously. Sections were scanned using the Ultra Fast Scanner (Philips, Eindhoven, The Netherlands), from which 10 images representative for the NOTCH3 accumulation observed in that mouse were obtained. To exclude a possible bias in image selection, a second, blinded observer was asked to obtain images independently (Additional file 1: Figure S1). ImageJ analysis was performed as follows: the image was converted into an 8-bit image, and filtered using the unsharp mask filter (radius 1, mask 0.60). Next, a threshold was set to a signal intensity of 150 to determine the NOTCH3-positive area. Within the NOTCH3-positive area, individual NOTCH3 particles were identified based on size and circularity (size = 0-30, circularity = 0.50-1.00). Finally, the NOTCH3 score was determined by quantifying the total area of the NOTCH3 positive particles within the image. The average of the three mice per time point was plotted and used for further statistical analysis.

### NOTCH3 immunohistochemistry and quantification in human material

We used paraffin embedded frontal lobe brain sections from three deceased CADASIL patients (I: female age 59, p.Arg153Cys; II: female age 57, p.Arg153Cys; III: male age 70, p.Cys446Phe) and three deceased controls with no known cerebrovascular disorders (I: male age 67, II: male age 58, III: male age 53). Sections were de-waxed, rinsed with ethanol and blocked with methanol/H_2_O_2_. After heat-induced antigen retrieval in 0.01 M citrate buffer pH 6, slices were washed three times with PBS, and incubated overnight at room temperature with a 1:1 cocktail of anti-NOTCH3^ECD^ (dilution 1:500) and anti-CD31 (Dako, Glostrup, Denmark; dilution 1:50). The following day, sections were washed and incubated for 1 hour at room temperature with a 1:1 cocktail of anti-rabbit Envision/HRP (Dako) and goat anti-mouse alkaline phosphatase (Vector Laboratories, Burlingame, CA, USA; dilution 1:25). Finally, sections were sequentially developed with 3,3′-diaminobenzidine solution and Vector Blue (Vector laboratories). Per individual, four images were taken at a 400× magnification on a Leica IM 500 microscope and analysed using ImageJ software. The vessel area was selected manually based on a positive CD31 staining. Within the vessel area, the NOTCH3 score was calculated using an intensity threshold of 100.

### Electron microscopy in *NOTCH3* transgenic mice

Brain tissue was fixed in 1.5 % glutaraldehyde and 1 % paraformaldehyde in 0.1 M cacodylate buffer, post-fixed in a solution of 2 % osmium tetroxide and 2 % potassium ferrocyanide, dehydrated and embedded in epon 812 (LX112). After selection of areas of interest on 1 μm toluidine stained sections, ultrathin sections were cut, contrasted with 3 % uranylacetate and Reynolds lead citrate and examined with a JEOL JEM-1011 electron microscope (Advanced Microscopy Techniques, Woburn, USA).

### Statistical analysis

Statistical analyses were performed using Graphpad Prism. Differences in NOTCH3 score between the two mouse strains at a given time point were analysed using the unpaired student’s *t*-test. Differences in NOTCH3 score between time points were analysed using One-Way ANOVA and Fishers least significant difference post-hoc analysis. Differences in slope (i.e. the rate of increase of the NOTCH3 score) over time were analysed using linear regression.

### Brain MRI of *NOTCH3* transgenic mice

Brain MRI was performed in 15 mice at 20 months of age; six TgN3^MUT^350 mice, five tgN3^WT^ mice, and four non-transgenic littermates. Mice were anesthetised by inhalation of 2 % isoflurane in a 1:1 mixture of oxygen and air. Respiration rate was monitored with a respiratory pad and kept between 50 and 80 respirations per minute by adjusting the isoflurane concentration. T2 weighted imaging was performed on a 7 Tesla Bruker Pharmascan using a 23 mm quadrature coil with the following parameters: TE = 12 ms, RARE factor = 8, effective TE = 48 ms, TR = 4 s, 8 averages. Field-of-view = 19x19 mm, matrix = 196x196, resulting in an in-plane resolution of 97 μm. Slice thickness = 0.5 mm, with 32 slices.

### Analysis of brain parenchyma in *NOTCH3* transgenic mice

After MRI, anesthetised mice were sacrificed using cardial perfusion with ice-cold phosphate buffered saline. One brain hemisphere was formalin fixated and paraffin embedded. Sections were stained with hematoxylin and eosin (H&E) to analyse the presence of infarctions, with Kluver Barrera Luxol fast blue to visualise myelin and with Perl’s iron to assess the presence of microbleeds. Astrogliosis was analysed using an anti-glial fibrillary acidic protein (GFAP) antibody (rabbit anti-GFAP, Dako; dilution 1:1000), which was incubated overnight at room temperature. As a secondary antibody, biotin labelled swine-anti Rabbit (Dako; dilution 1:600) was used, this was incubated 1 hour at room temperature. Finally, sections were incubated with avidin-biotin complex (Vectastain ABC-Elite Kit, Vector Lab, Burlingame, USA) for 30 minutes at room temperature and developed in 3,3′-diaminobenzidine solution. The detection of macrophages and myelin was performed using the Animal Research Kit peroxidase (Dako). Biotinylated primary antibodies against CD68 (anti-CD68 clone KP-1, Dako; dilution 1:1000) and myelin proteolipid protein (anti-PLP, clone plpc-1, Serotec, Kidlington, UK; dilution 1:500) were incubated overnight after heat-induced antigen retrieval in 0.01 M EDTA pH 8.0. The following day, sections were incubated with HRP- conjugated streptavidin for 30 minutes at room temperature and developed in 3,3′-diaminobenzidine solution.

## Results

### Four mutant human *NOTCH3* transgenic mouse strains with distinct *NOTCH3* expression levels

Using qPCR analysis on RNA isolated from brain of 10-week old mice, we found that the four mutant *NOTCH3* p.Arg182Cys transgenic mouse strains (tgN3^MUT^) had human *NOTCH3* expression levels of 100, 150, 200 and 350 %, respectively, compared to endogenous mouse *Notch3* expression (Fig. 1c). Human *NOTCH3* expression in the lowest expressing tgN3^MUT^ strain, was comparable to that in the wild-type strain (tgN3^WT^), i.e. ~100 %. RT-PCR analysis with multiple *NOTCH3* primer sets spanning the complete transcript, showed that the complete human *NOTCH3* cDNA was present in the transgenic transcript (Additional file 1: Figure S2). There was no difference in endogenous mouse *Notch3* expression between transgenic and non-transgenic mice (Additional file 1: Figure S3).

### Age at onset of vascular NOTCH3 protein accumulation correlates with *NOTCH3* expression levels

**Fig. 2:**
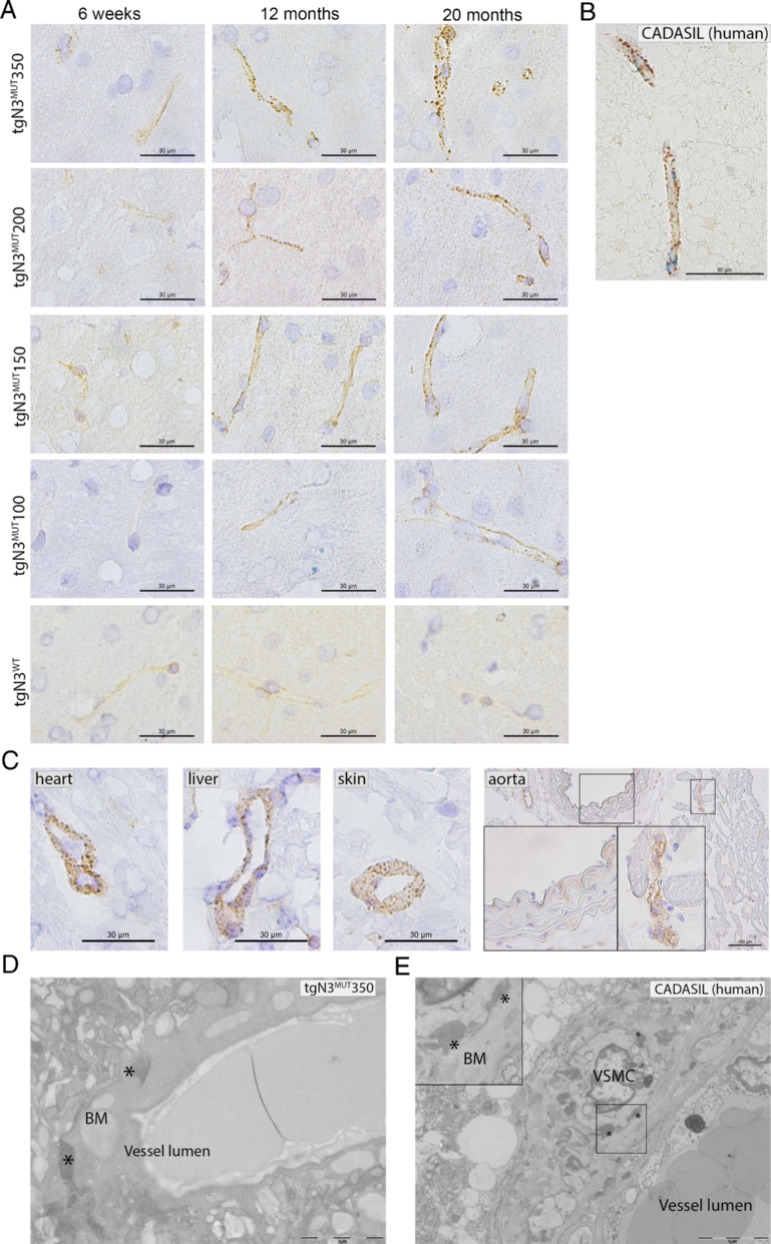
Vascular NOTCH3 protein accumulation and GOM deposits in transgenic human *NOTCH3* p.Arg182Cys mice. **a** NOTCH3 immunostaining on brain sections of human *NOTCH3* transgenic mice. All four tgN3^MUT^ mouse strains developed a characteristic granular NOTCH3 staining pattern in the brain vasculature. TgN3^WT^ mice showed only a weak, diffuse NOTCH3 staining pattern, which did not increase with age (comparable to non-transgenic litter-mates, data not shown). The NOTCH3 accumulation load in the tgN3^MUT^ strains correlates well with the *NOTCH3* expression level and increases with age; in tgN3^MUT^350 mice, first granular staining is already visible at 6 weeks of age; at 20 months of age nearly the whole vessel wall is packed with big granular NOTCH3 deposits. In the strains with a lower *NOTCH3* expression level, the NOTCH3 accumulation starts at a later age and the granular deposits remain smaller. **b** Positive NOTCH3 staining in a brain vessel of a CADASIL patient. **c** NOTCH3 immunostaining of extra-cerebral arteries of 20-month-old tgN3^MUT^350 mice showing clear granular NOTCH3 staining in vessels of the heart, liver and skin. The aortic wall shows a diffuse and faint NOTCH3 staining pattern comparable to that seen in non-transgenic littermates, whereas the smaller vessels around the aorta do show characteristic granular NOTCH3 staining. **d** Electron microscopy on brain vessels from 12-month-old tgN3^MUT^350 mice shows characteristic electron dense deposits reminiscent of granular osmiophilic material (GOM). GOM deposits were first seen at 5–6 months of age. **e** Electron microscopy on brain tissue from a deceased CADASIL patient shows pathognomonic GOM deposits, adjacent to the basement membrane surrounding the VSMCs. * = granular osmiophilic material (GOM), BM = basement membrane, VSMC = vascular smooth muscle cell

To analyse the presence and onset of a CADASIL vascular phenotype, NOTCH3 immunohistochemistry was performed on brain slices from mice between the ages of 4 weeks and 20 months. This showed that all tgN3^MUT^ strains developed cerebrovascular NOTCH3 accumulation, as seen by a positive, granular NOTCH3 staining of the vessel wall (Fig. 2a), similar to that which is seen in CADASIL patients (Fig. 2b). There was a considerable difference in age at onset of positive NOTCH3 staining per mouse strain, ranging from 6 weeks in tgN3^MUT^350 mice to 12 months in tgN3^MUT^100 mice. The age at onset directly correlated with the level of human *NOTCH3* RNA expression for all four tgN3^MUT^ strains i.e. the higher the *NOTCH3* RNA expression level, the earlier the onset of NOTCH3 accumulation (Table 1). Furthermore, in each mutant strain, the positive NOTCH3 immunostaining became progressively more intense and granular with age (Fig. 2a). The individual granular NOTCH3 deposits increased not only in number, but also in size. This was most prominent in mice with the highest *NOTCH3* RNA expression level (tgN3^MUT^350), in which the NOTCH3 protein accumulation progressively evolved to a vessel wall packed with intense and big granular NOTCH3 deposits at age 20 months. Characteristic granular NOTCH3 staining was also present in arterioles of the heart, liver, kidney, skin and tail, but not in the aorta (Fig. 2c, data not shown). Overall, the NOTCH3 accumulation observed in the extra-cerebral arterioles was less pronounced than in the brain. Electron microscopy of brain arterioles revealed characteristic electron dense deposits within the basement membrane (Fig. 2d) reminiscent of GOM deposits seen in CADASIL patients (Fig. 2e). Neither GOM nor increased cerebrovascular NOTCH3 staining was found in tgN3^WT^ mice at 20 months of age (Fig. 2a). Taken together, these analyses show that transgenic human *NOTCH3* p.Arg182Cys mice develop an early and progressive systemic arteriopathy which closely resembles the vascular pathology seen in CADASIL patients, with age-at-onset correlating with the respective levels of mutant human *NOTCH3* expression.

**Table 1:** The *NOTCH3* RNA expression level correlates with the age at onset of cerebrovascular NOTCH3 protein accumulation

Mouse strain	*NOTCH3* expression level^a^	Age at onset NOTCH3 accumulation^b^
tgN3^MUT^ 350	350 %	6 weeks
tgN3^MUT^ 200	200 %	3 months
tgN3^MUT^ 150	150 %	5 months
tgN3^MUT^ 100	100 %	12 months

^a^ mRNA *NOTCH3* expression levels relative to endogenous mouse *Notch3* expression levels ^b^ first sign of positive, granular NOTCH3 staining in brain vessels, as determined by an experienced neuropathologist (S.v.D)

### Development of the NOTCH3 score, a quantitative biomarker for CADASIL

**Fig. 3:**
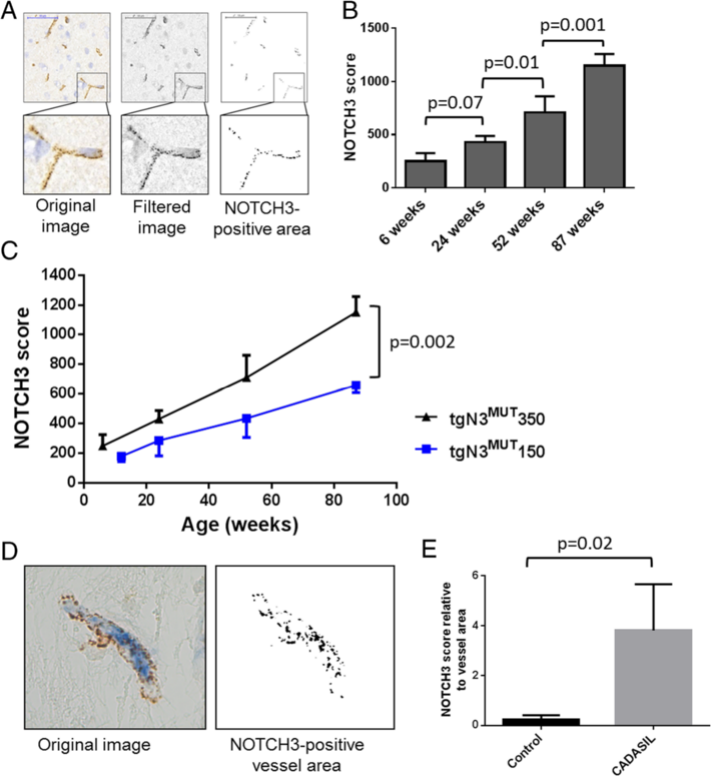
Quantitative analysis of vascular NOTCH3 protein accumulation in transgenic human *NOTCH3* p.Arg182Cys mice and in brain tissue of CADASIL patients. **a** ImageJ processing of NOTCH3-immunostained brain sections of tgN3^MUT^350 mice. The images were filtered to reduce background signal and a standardised threshold was applied to determine the NOTCH3- positive area composed of individual granular NOTCH3 deposits, resulting in the NOTCH3 score. **b** Quantitative analysis of NOTCH3 accumulation in tgN3^MUT^350 mice. The NOTCH3 score shows an age-dependent increase and allows for a sensitive discrimination between age groups (One-Way ANOVA, Fishers least significant difference). **c** Validation of the NOTCH3 score in tgN3^MUT^150 mice, also showing an age-dependent increase. At each time point, the score is lower in tgN3^MUT^150 than in tgN3^MUT^350 mice (unpaired *t*-test), reflecting the correlation between *NOTCH3* RNA expression and NOTCH3 protein accumulation. Data represent the average +/− SD of the three mice analysed per time point. **d** ImageJ analysis of human brain sections double stained with NOTCH3 and CD31. The vessel area was selected based on the staining with the endothelial cell marker CD31, and within this area, the NOTCH3 score was determined. **e** CADASIL patients show a significantly higher NOTCH3 score than age-matched controls. (unpaired *t*-test) Data represent the average +/− SD of three CADASIL patients and three control individuals

As we observed such an early and clear age- and *NOTCH3* expression level- dependent vascular NOTCH3 accumulation load, we set out to objectify this by developing a quantitative measure for NOTCH3 staining. This was accomplished by capturing and measuring the surface area of CADASIL specific granular NOTCH3 deposits within brain sections using ImageJ software (Fig. 3a), which we called the ‘NOTCH3 score’. This quantification was first performed in tgN3^MUT^350 mice, which clearly showed that the NOTCH3 score increased with age, confirming our qualitative observations (Fig. 3b). Next, we validated the NOTCH3 score in a second mouse strain, tgN3^MUT^150, in which the same age-dependent increase in the NOTCH3 score was seen. At each time-point, the NOTCH3 score was lower for the tgN3^MUT^150 mice compared to the tgN3^MUT^350 mice, reflecting the correlation between *NOTCH3* expression level and NOTCH3 accumulation load (score at age 20 months: 659 ± 51 *vs.* 1150 ± 107, *p* = 0.002) (Fig. 3c). Furthermore, progression of NOTCH3 accumulation was slower in tgN3^MUT^150 mice compared to tgN3^MUT^350 mice, as shown by a significant difference in the slope of the NOTCH3 scores between the two mouse strains (11.1 ± 0.5 *vs.* 6.2 ± 0.3, *p* = 0.002). Finally, we tested the approach in brain sections of three unrelated CADASIL patients. Measurement of the NOTCH3 accumulation load using the NOTCH3 score was technically feasible in human tissue (Fig. 3d) and showed a significantly higher NOTCH3 score in patients than in controls (score 3.81 ± 1.85 *vs.* 0.24 ± 0.17, *p* = 0.02) (Fig. 3e).

### No clear brain parenchyma phenotype in tgN3^MUT^ mice

Finally, to determine whether we could correlate the NOTCH3 score to a brain phenotype in tgN3^MUT^ mice, we performed brain MRI and histopathology in mice aged 20 months (Additional file 1, Figure S4, S5). In two of the six tgN3^MUT^350 mice, hyperintensities were seen on T2 weighted images, cranial to the corpus callosum and around the ventricles in the frontal lobe. However, similar hyperintensities were found in one non-transgenic littermate. Histopathological examination did not show any signs of astrogliosis or white matter lesions in the mutant mice. The absence of consistent and specific brain abnormalities in tgN3^MUT^ mice prohibited the testing of a potential correlation between NOTCH3 score and brain phenotype.

## Discussion

To facilitate the testing of pre-clinical therapeutic interventions for CADASIL, we generated a translational, human genomic *NOTCH3* transgenic mouse model with an early vascular phenotype, and developed a biomarker in this model. The mutant mice recapitulate the CADASIL vascular phenotype with early onset and progressive cerebrovascular NOTCH3 accumulation and GOM deposits in arterioles. The respective *NOTCH3* RNA expression levels in the four mutant mouse strains correlate strongly and consistently with the age at onset and progression of NOTCH3 protein accumulation, with the highest expressing mouse strain developing vascular NOTCH3 accumulation as early as 6 weeks of age. The quantitative biomarker we developed, the NOTCH3 score, allows for a sensitive and objective measure of NOTCH3 accumulation, which can therefore be used for pre-clinical testing of therapeutic strategies aimed at delaying or reversing NOTCH3 accumulation.

Cerebrovascular NOTCH3 accumulation was selected as a potential biomarker because it was consistently and specifically found in tgN3^MUT^ mice, and showed an early age at onset and clear progression. Also, NOTCH3 accumulation is a plausible surrogate marker for CADASIL because it is universally present in the cerebrovasculature of CADASIL patients and is believed to play an important role in disease pathophysiology [22]. Because of the lack of consistent brain abnormalities in our mice, we were unable to correlate the NOTCH3 score to a brain phenotype. However, an early age at onset of NOTCH3 accumulation has previously been found to be associated with the development of brain parenchymal damage in mice [9]. Moreover, age is one of the most important predictors of CADASIL disease severity and progression [14, 23], implicating that the age-dependent increase in NOTCH3 score is a relevant surrogate marker for disease progression. Sample size calculations we performed show that the NOTCH3 score is a feasible biomarker for pre-clinical therapeutic studies, as an effect on NOTCH3 accumulation can be assessed in relatively small groups of mice. For example, treatment of 7 mice allows for the detection of a 50 % effect on the progression of the NOTCH3 score, when treating from 6 to 24 weeks of age.

We found that the NOTCH3 score can also be measured in the cerebrovasculature of deceased CADASIL patients. Evidently, a NOTCH3 score in brain sections is not a feasible biomarker in clinical trials. However, vascular NOTCH3 accumulation has been extensively demonstrated in skin biopsies of CADASIL patients and is detectable decades before the onset of clinical symptoms [18, 19]. In a single family study, an age-dependent increase in GOM deposits in skin biopsies was found up to 50 years of age [24]. Although previous studies did not find a correlation between skin biopsy NOTCH3 immunostaining and disease severity, these studies were limited by a qualitative assessment of the NOTCH3 staining intensity [18, 19]. Whether such a correlation can be established using our quantitative NOTCH3 score, will have to be assessed in future prospective studies.

This novel CADASIL mouse model is especially suitable for testing therapeutic strategies for a number of reasons. The presence of the human *NOTCH3* gene in our mouse model allows for testing compounds specifically directed at human *NOTCH3*, thereby avoiding an additional hurdle in the translation from pre-clinical to clinical trials. The fact that we used a genomic *NOTCH3* construct allows for testing therapeutic interventions that target mutant *NOTCH3* at the genomic or (pre-) mRNA level. Such interventions, for example using antisense oligonucleotides to reduce or modify mutant NOTCH3 protein, are being developed in our lab (Rutten et al., unpublished, patent no. WO 2010085151 A2). Another practical advantage is that in this early onset model, treatment can be initiated at an early age.

## Conclusions

In conclusion, we developed a novel, unique human *NOTCH3* transgenic mouse model and a NOTCH3 score which is a robust and sensitive biomarker for CADASIL. This translational model is ideally suited for pre-clinical testing of therapeutic strategies aimed at delaying or reversing NOTCH3 protein accumulation.

## Additional file

Additional file 1:
**Supplemental materials.** (DOCX 2768 kb)

## Abbreviations

CADASIL: Cerebral Autosomal Dominant Arteriopathy with Subcortical Infarcts and Leukoencephalopathy
NOTCH3^ECD^: Extracellular domain of the NOTCH3 protein
GOM: Granular osmiophillic material
VSMCs: Vascular smooth muscle cells
MRI: Magnetic resonance imaging
