# Supplementary material for: The NOTCH3 score: a pre-clinical CADASIL biomarker in a novel human genomic NOTCH3 transgenic mouse model with early progressive vascular NOTCH3 accumulation
Source: Acta Neuropathol Commun. 2015 Dec 29;3:89. doi: 10.1186/s40478-015-0268-1 (PMC4696336; doi:10.1186/s40478-015-0268-1)
Supplement: Additional file 1: — Supplemental materials. (DOCX 2768 kb) [file 40478_2015_268_MOESM1_ESM.docx]

**Additional file 1**

*Genotyping primers*

| **Primer** | **Sequence** |
| --- | --- |
| HuNOTCH3ex 4F | 5’-CTGACTGCTCCCTGCCAGAT-3’ |
| HuNOTCH3ex 4R | 5’-TAAGTGAGGTCGCCACTCTG-3’ |
| SYDE1Dex2 F | 5’-AATTGCACAGGGCTGCTC-3’ |
| SYDE1Dex2 R | 5’-AGGTACCCCAGGCCCTAAA-3’ |
| ILVBLex2 F | 5’-AGGGGCGCGATTTCTTC-3’ |
| ILVBLex2 R | 5’-AGGCCCCTCCCTCTTTG-3’ |
| EPHX3ex5 F | 5’-GTAGGGGTGCAGGTGTATGG-3’ |
| EPHX3ex5 F | 5’-TGGCTTCAGTGCCCAGC-3’ |

*RT-PCR primers*

| **Primer** | **Sequence** |
| --- | --- |
| HuNOTCH3ex 2F | 5’-GTGTGCAAATGGAGGTCGT-3’ |
| HuNOTCH3ex 4R | 5’-TAAGTGAGGTCGCCACTCTG-3’ |
| HuNOTCH3ex 14F | 5’-GCGATGGAATGGGTTTCCA-3’ |
| HuNOTCH3ex 16R | 5’-GCCAGGTTGGTGCAGATACCATGA-3’ |
| HuNOTCH3ex 30F | 5’-TCTCCGACCTGATCTGCCAG-3’ |
| HuNOTCH3ex 32R | 5’-TTGAGCAGGGCCAAAGTGGCT-3’ |
| HuNOTCH3ex 32F | 5’-AGCCACTTTGGCCCTGCTCAA-3’ |
| HuNOTCH3ex 33R | 5’-GTTGATCCAGCAAGCGCACG-3’ |
| HuNOTCH3ex 32-33F | 5’-GGACATGCAGGATAGCAAGGAG-3’ |
| HuNOTCH3ex 33R_3UTR | 5’-CGGTCACGCTGCAAGGCAAGG-3’ |

*qPCR primers*

| **Primer** | **Sequence** |
| --- | --- |
| HuNOTCH3ex 7F | 5’-GGACGAGTGCTCTATCGGC-3’ |
| HuNOTCH3ex 9R | 5’-GTTCCTGTGAAGCCTGCCATA-3’ |
| MsNotch3ex 6F | 5’- TTCTACTGTGCCTGCCCT-3’ |
| MsNotch3ex 8R | 5’-CACACCGACCCAAATGTTCA-3’ |
| MsGAPDH F | 5’-TGCACCACCAACTGCTTAGC-3’ |
| MsGAPDH R | 5’-GGCATGGACTGTGGTCATGAG-3’ |

**Table S1 Primers used for mouse genotyping and *NOTCH3* expression analysis.**

**Figure S1** **Validation of NOTCH3 protein quantification in transgenic human *NOTCH3* mice.** No differences in NOTCH3 score were seen upon image selection by two independent observers (score tgN3^MUT^350: 1572 ±448 vs. 1394 ±318 p=0.54, unpaired *t*-test). Data represents the mean ±SD of 4 images.


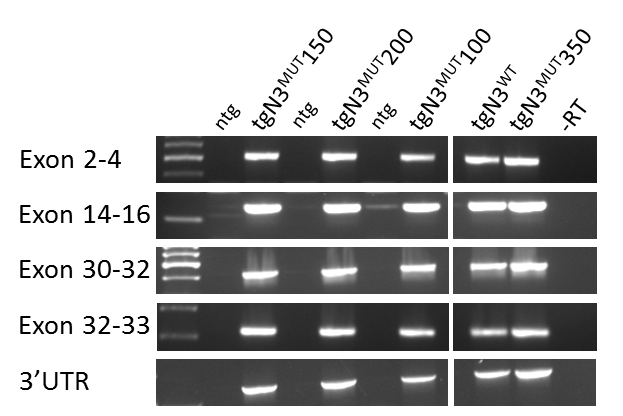


**Figure S2 *NOTCH3* RNA expression in transgenic human *NOTCH3* mice.** RT-PCR analysis using primers spanning the *NOTCH3* gene showed that the complete *NOTCH3* transcript is expressed in all transgenic mouse strains.

**Figure S3 Endogenous *Notch3* RNA expression in transgenic human *NOTCH3* mice.** qPCR analysis shows similar *Notch3* expression levels in brain tissue of transgenic and non-transgenic mice. **
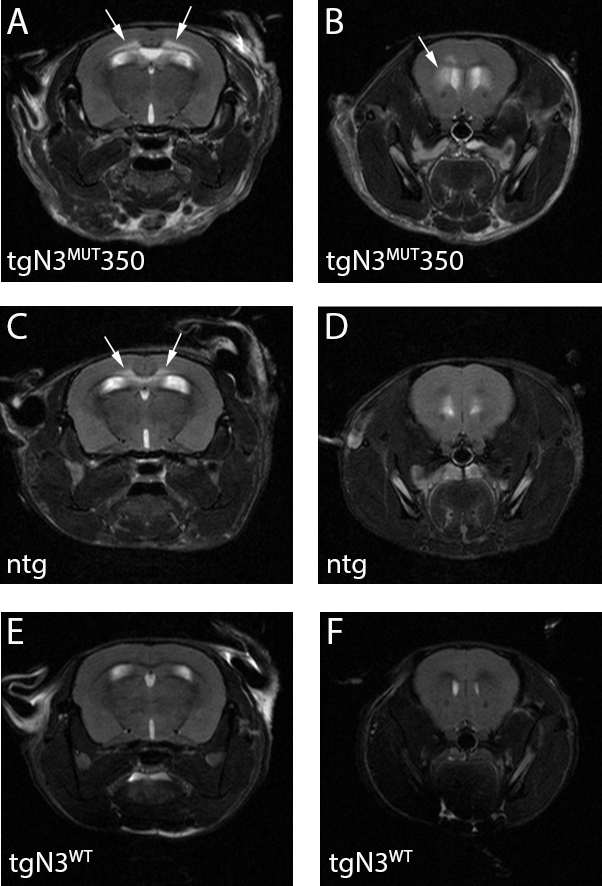
**

**Figure S4 Brain MRI of transgenic human *NOTCH3* p.Arg182Cys mice.** **(A-F)** T2 weighted brain MRI images of mice aged 20 months. Six tgN3^MUT^ mice, five tgN3^WT^ mice and four non-transgenic littermates were analysed. Brain MRI abnormalities (indicated by the arrows) were detected in two of the six tgN3^MUT^350 mice, and in one of the four non-transgenic mice. All mice with hyperintensities also had enlarged ventricles, which is a common finding in C57Bl/6J mice (The laboratory mouse, 2^nd^ edition) **(A-B)** Representative pictures of one of the two tgN3^MUT^350 mice with brain MRI abnormalities. T2 weighted brain MRI showed hyperintensities cranial to the corpus callosum, and in the frontal lobe around the ventricles. **(C-D)** Similar hyperintensities, although less severe, were seen in one of the non-transgenic littermates. **(E-F)** No MRI abnormalities were seen in tgN3^WT^ mice.

**
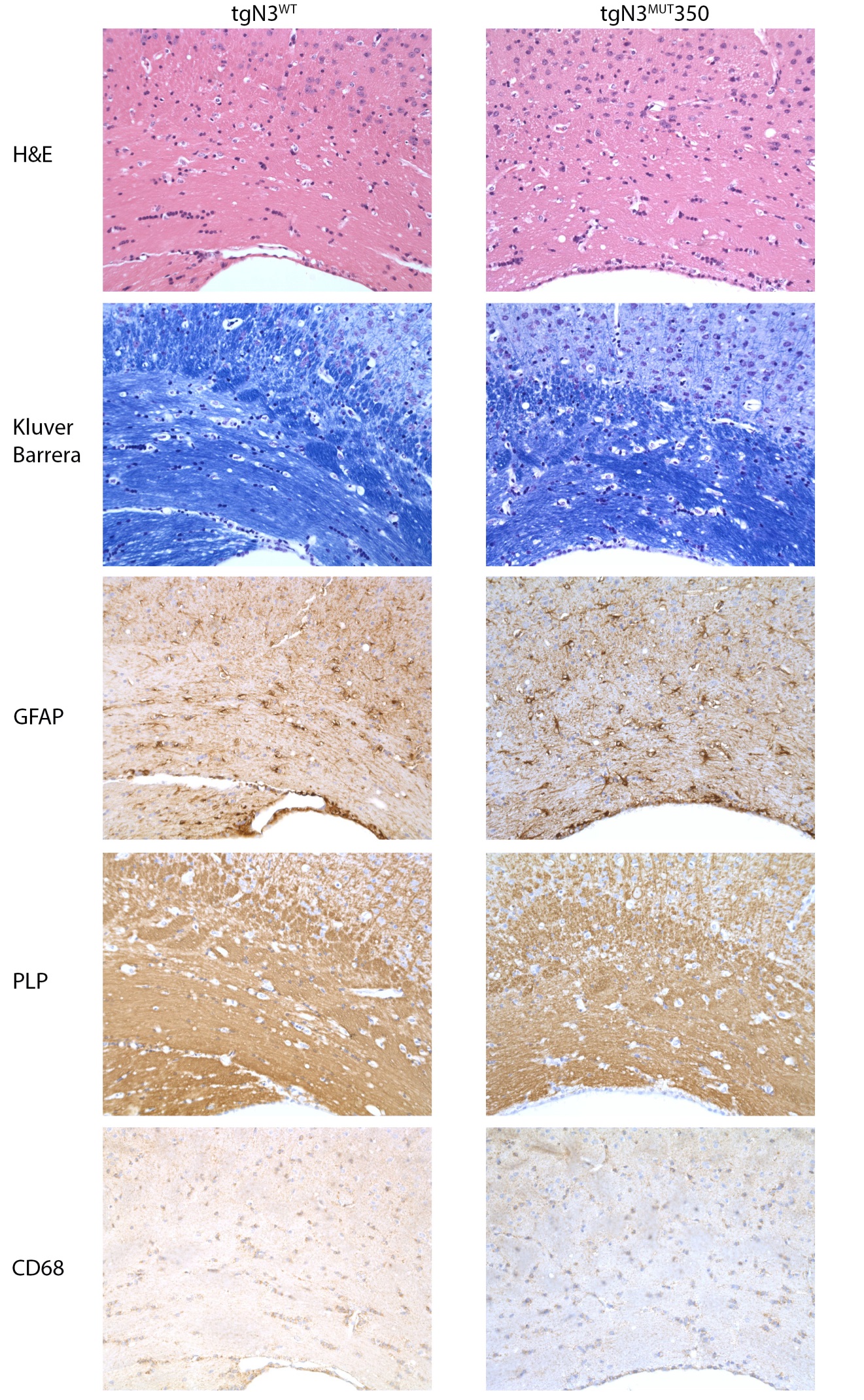
**

**Figure S5 Brain parenchyma stainings of tgN3^MUT^350 and tgN3^WT^ mice.** No consistent differences were seen for any of the stainings analysed. Moreover, no histopathologic substrate for the observed MRI abnormalities was seen. All images are obtained at a 200x magnification.
